# Supplementary material for: How can school help victims of violence? Evaluation of online training for European schools’ staff from a multidisciplinary approach
Source: PLoS One. 2022 Aug 15;17(8):e0272872. doi: 10.1371/journal.pone.0272872 (PMC9377607; doi:10.1371/journal.pone.0272872)
Supplement: S1 Fig — As expected, some categories are more frequent in individual interviews (e.g., category 4 in interview 2), since each individual was most sensitive about particular issues or proposals. However, most categories are present in all interviews, showing that the system tries to balance the presence of common concepts across interviews (similarity) and respects the particularity of each discourse (diversity). (DOCX) [file pone.0272872.s001.docx]

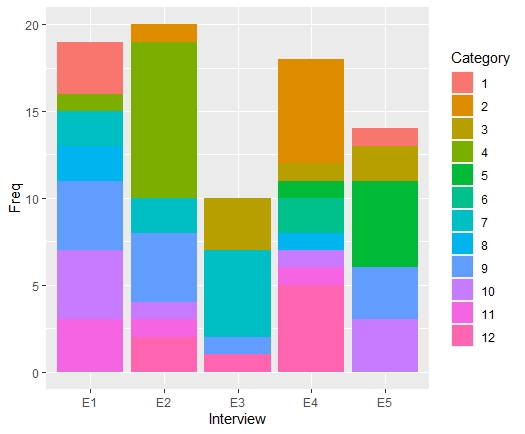


**S1 Fig*.* Distribution of categories across interviews.** As expected, some categories are more frequent in individual interviews (e.g., category 4 in interview 2), since each individual was most sensitive about particular issues or proposals. However, most categories are present in all interviews, showing that the system tries to balance the presence of common concepts across interviews (similarity) and respects the particularity of each discourse (diversity).
