# Supplementary material for: How can school help victims of violence? Evaluation of online training for European schools’ staff from a multidisciplinary approach
Source: PLoS One. 2022 Aug 15;17(8):e0272872. doi: 10.1371/journal.pone.0272872 (PMC9377607; doi:10.1371/journal.pone.0272872)
Supplement: S1 Table — (DOCX) [file pone.0272872.s002.docx]

**S1 Table. Contingency table for study II categories and sociodemographic variables**

|  | Actions related to report | Actions related to detection | Everyday actions | Actions involving a whole school approach | Total | Fischer’s exact test |
| --- | --- | --- | --- | --- | --- | --- |
| *Gender* |  |  |  |  |  | .77 |
| Men | 0 | 2 | 3 | 2 | 7 |  |
| Women | 4 | 11 | 16 | 8 | 39 |  |
| *Age* |  |  |  |  |  | **14.49** |
| 21-34 y.o. | 4 | 3 | 9 | 2 | 18 |  |
| 35-44 y.o. | 0 | 6 | 8 | 2 | 16 |  |
| 45-54 y.o. | 0 | 3 | 1 | 5 | 9 |  |
| 55+ y.o. | 0 | 1 | 1 | 1 | 3 |  |
| *Nationality* |  |  |  |  |  | 15.60 |
| Irish | 0 | 0 | 5 | 0 | 5 |  |
| Italian | 0 | 2 | 0 | 0 | 2 |  |
| Polish | 0 | 3 | 2 | 4 | 9 |  |
| Spanish | 4 | 7 | 12 | 6 | 29 |  |
| Non-European | 0 | 1 | 0 | 0 | 1 |  |
| *Level of studies* |  |  |  |  |  | 5.92 |
| High-School | 0 | 1 | 0 | 0 |  |  |
| Tertiary | 1 | 2 | 2 | 0 |  |  |
| University | 3 | 10 | 17 | 10 |  |  |

*Note.* Verbatims that could be assigned to more than one category were excluded from this analysis. Statistically significant results of Fischer’s exact test at *p* <.05 level are bolded. Given that some cells do not reach minimum frequency, we focused on checking the distribution of categories based on the analyzed variables. Distribution suggest that categories are evenly present among males and females, level of studies or occupation. Interestingly, participants from Ireland focused on describing everyday actions. This tendency may arouse from the fact that in Ireland training to detect and report Child Abuse and Neglect are mandatory (McGarry & Buckley, 2013) and studies performed in this country have highlighted the importance of daily actions in order to give support and protect children against violence from school (Nohilly, 2019). Italian school staff focused on actions related to detection. Respondents from Poland described action in almost every category. Participants from Spain, where studies have shown that report potential child victimization is particularly challenging (Greco et al., 2020) were the only group to describe actions related to reporting.
