# Supplementary material for: How can school help victims of violence? Evaluation of online training for European schools’ staff from a multidisciplinary approach
Source: PLoS One. 2022 Aug 15;17(8):e0272872. doi: 10.1371/journal.pone.0272872 (PMC9377607; doi:10.1371/journal.pone.0272872)
Supplement: S2 Table — (DOCX) [file pone.0272872.s003.docx]

**S2 Table. Links between findings of study I and study II**

| Category | Actions | Type of action |
| --- | --- | --- |
| Empathy | *To be more mindful of students home situation & their feelings. To develop positive relationships with students. To have students develop a sense of trust in me.* | Everyday |
|  | *I feel more confident about the climate that I want to create in my classroom: respect, acceptance, collaboration and affection. Although my colleagues do not agree and see it as permissive, it has empowered me.* | Everyday |
|  | *Talk to them, build trust.* | Everyday |
|  | *Active listening and a climate of security and trust* | Everyday |
|  | *(…) and boldly undertake interventions (by building a therapeutic alliance and an atmosphere conducive to talking about children’s concerns). I will think about the form of psychoeducational classes that raise (in a manner adapted to the students’ abilities) the issue of safety also in terms of my own body* | Everyday |
| Observation | *A more in-depth look at the symptoms of children's behaviour, greater attention to peer relationships, looking at school procedures (adequacy, readability, usefulness)* | Detection |
|  | *Pay more attention to the psychological cues children may show after experiencing child abuse.* | Detection |
|  | *Using the ability to spot early signs of violence and interventions* | Detection |
|  | *Observe more carefully the behaviour or symptoms of my students at recess. Find out if measures against cyberbullying are included in our protocol* | Detection |
|  | *More attention to the signals reported by children* | Detection |
|  | *I’ll take into account some observation guidelines to detect risks* | Detection |
|  | *The statistics shocked me, now I will look at students much more closely, keeping in mind the memorized indicators* | Detection |
|  | *The look, already more sensitive and attentive* | Everyday |
| Adultcentrism | *Ensure by my classroom behaviour that I am exhibiting willingness and availability to listen to students if and when needed. Sometimes we appear so busy students do not wish to disturb a working teacher.* | Everyday |
|  | *When there are suspicions of abuse or mistreatment, report it, do not investigate its veracity.* | Report |
|  | *The way to manage the information received directly from children, how to legally deal with situations, what steps to take, what responsibilities must be assumed both individually and by the educational centre* | Report |
|  | *Listen more to the children* | Everyday |
|  | *To be more mindful of students’ home situation & their feelings. To develop positive relationships with students. To have students develop a sense of trust in me.* | Everyday |
| Giving value to emotions | *The introduction of a calming space for kids to calm themselves* | Everyday |
|  | *To be more mindful of students’ home situation & their feelings. To develop positive relationships with students. To have students develop a sense of trust in me.* | Everyday |
|  | *The look, already more sensitive and attentive* | Everyday |
|  | *I will certainly pay more attention to even the small emotional changes of my pupils* | Detection |
|  | *I will include some of the aspects mentioned in the affective and sexual education workshops in primary school. And I will continue to learn more about it because I consider it very important and there were some aspects that I did not know about the action* | Everyday |
| Breaking the silence | *In my school we have been training in recent years on these topics. We know the protocols of our autonomous community and the forms of action are included in the centre’s documents. Until this course we had not talked about CSA, but I gave a talk to my classmates on this topic (on which I trained as much as possible) and we are waiting to include it too* | At school level |
|  | *Ensure by my classroom behaviour that I am exhibiting the willingness and availability to listen to students when needed. Sometimes we appear so busy students do not wish to disturb a working teacher.* | Everyday |
| Actions to promote disclosure | *The way to manage the information received directly by the children, how to legally deal with situations, what steps to take, what responsibilities must be assumed both individually and by the education centre* | Report |
|  | *Talk to them, build trust* | Everyday |
| Institutional work | *By extending training to all school employees I will promote among educators the establishment of anti-bullying codes with children* | At school level |
|  | *In my current workplace we have clear actions regarding abuse, mistreatment and harassment, and according to what I have been able to verify in the course, we are acting correctly.* | At school level |
|  | *To insist more that the school’s management team extends the training to teaching and service staff.* | At school level |
|  | *By working with the advice and coordination of interdisciplinary teams in the educational field, I consider that the course offers specific tools and information to act from prevention and intervention, elements that can be addressed with the rest of the centre’s team and implemented from training sessions or workshops. Reflection activities also seem useful to me to share with professionals in the workplace and enable the exchange of experiences and strategies to develop inside and outside the classroom.* | At school level |
| School as a refuge | *Active listening and a climate of security and trust* | Everyday |
|  | *Take another perspective and security into account in decision-making* | Everyday |
|  | *Raising awareness of responsibility for the well-being of students. Talking to other teachers, sharing news* | At school level |
| Isolation from peers | *A more in-depth look at the symptoms of children's behaviour, greater attention to peer relationships, looking at school procedures (adequacy, readability, usefulness)* | Detection |
|  | *More work on social relations in the classroom* | Everyday |
|  | *Rewards for pro social behaviour* | Everyday |
|  | *More activities to prevent peer violence and build relationships during parental hours* | Everyday |
| Family dynamics | *To be more mindful of students’ home situation and their feelings* | Everyday |
|  | *More activities to prevent peer violence and build relationships during parental hours* | Everyday |
| Excessive compliance | *Ensure by my classroom behaviour that I am exhibiting willingness and availability to listen to students if and when needed. Sometimes we appear so busy students do not wish to disturb a working teacher.* | Everyday |
| Work with perpetrators | *I will review in greater depth possible cases of bullying in the classroom* | Detection |
|  | *Rewards for pro-social behaviour* | Everyday |
|  | *Rewards for social behaviour* | Everyday |
