# Supplementary material for: How can school help victims of violence? Evaluation of online training for European schools’ staff from a multidisciplinary approach
Source: PLoS One. 2022 Aug 15;17(8):e0272872. doi: 10.1371/journal.pone.0272872 (PMC9377607; doi:10.1371/journal.pone.0272872)
Supplement: Attachment — Submitted filename: Training school staff in victimization - feedback2.pdf [file pone.0272872.s005.pdf]

**PLOS ONE**

**Manuscript Number:** PONE-D-21-22475

**Tittle:** How can school help victims of violence? Evaluation of online training for European schools' staff from a multidisciplinary approach

The idea of your article is of great importance knowing the number of digital courses and online resources that are developed to support teachers in their encounters with children experiencing violence without being evaluated. We therefore do not know much about these programs' effectiveness, availability for teachers, and to what extend they meet the teachers' and the victims' needs. Your design is creative and well explained. The article would benefit from a theoretical framework to provide a deeper analysis of the data and a more thorough discussion. For the meantime, the discussion part is more or less descriptive than discussive.

**I recommend this article for publication with major revisions.**

I have organized my feedback in the following table according to the different parts of the article:

| **Part of the article** | **Line number** | **Citation from the article** | **Comment** |
|---|---|---|---|
| Abstract | | | No comments |
| Introduction | 29 | … forms that are specific to their age group | Elaborate a bit more on that. What do you mean? Maybe provide some examples. |
| | 73-81 | | I recommend adding to this part the doctoral study of Morrison which is specifically on the disclosure of children who experience sexual abuse:<br><br>Morrison, S. E. (2016). *Legal disclosure of childhood sexual abuse: what can professionals tell us?* [Doctoral dissertation]. University of Glasgow. |
| | 92 | It also proposes a module about resilience… | Can you explain a bit more what you mean with that? This part of information is too short. |

| | | | |
|---|---|---|---|
| | 93 | …and additional material to use in school. | Such as? Could you provide some more information? |
| | 98-99 | To explore how victims of sexual abuse during childhood remember the school's role in their lives at the time, to try to find out what helped them and what made it harder for them. | School systems, teacher education, perception of children etc. are in continuous change. What adult informants tell about their past experiences ought to be more problematized and not just taken for granted knowing that the school at informants' time have changed from the time of their experience to the time of this research.<br>Its important to discuss this either under method or limitation. It would be also of importance also to discuss what would the research have gained if the informants where to be children at the time of the research, and why not recruiting children who have experienced sexual abuse and were living under safe conditions during the research. |
| Materials and methods | 117 | SAVE was created by a partnership of five European entities based in… | Maybe it should be written four? Since only 4 partnership entities are listed below: Ireland, Italy, Spain, Poland. |
| | 124-125 | A challenging aspect of SAVE was that it had to be applicable across Europe, so we had to take into 124 account the particularities of each region, such as the official languages, the different procedures to 125 | What about the teacher education between these countries?<br><br>Teacher education will have a great influence on what and how much teachers know about this topic and how they react. This can be very different between countries so as between universities within the same country. |

| | | | |
|---|---|---|---|
| | | report child victimization or the required training for school staff members in each country. | Teachers' attitude and understanding of their role can be of great importance to how they react towards children's disclosure.<br><br>See for example:<br>Ellis, G. (2018). Containment and denial: Raising awareness of unconscious processes present when teachers are working with children and families where there is domestic abuse. *Educational Psychology in Practice*, *34*(4), 412–429. |
| | 138 | All of them were Spanish and… | I understand that for convenience informants where all female Spanish.<br><br>What about males?<br><br>However, would not the research have benefitted from recruiting informants from other parts of Europe also to test out the availability and applicability of this program to European countries other than the countries represented by the 4 partnership entities? And nevertheless, also males?<br><br>This ought to be discussed as part of the limitations of this research.<br><br>What the age range of the informants of this research? |
| | 149 | Most were highly educated females. | This says little about the informants.<br>Are they educated in different fields, or all are graduates from teacher education? |

| | | | |
|---|---|---|---|
| | 216 | The principles of conventional content analysis… | According to which approach? |
| | 240 | To store, manage, and organize the data, all transcripts were uploaded to the Open Source R Software. | Can the authors elaborate more about the use of the R software? |
| | 269 | All analysis were performed using R | Its unclear. Needs more elaboration. |
| Results | 271-303 | | Consider another presentation for the results that is more reader friendly. Maybe a table? |
| Discussion | 311 | Previous studies already noted the lower performance… | The authors mention “previous studies” and refers to only one study. However, lower performance it is not always the case. Some children who experience domestic violence might do well at school as a coping strategy. See for example:<br><br>Selvik, S. (2020). School strategies of children with multiple relocations at refuges for abused women. *Scandinavian Journal of Educational Research*, *64*(2), 227-241. |
| | 323 | …importance of a private, safe space to share their feelings… | As Morrison (2016) report in her research “the opportunity to tell”. |
| | 341-342 | It could also be due to the fact that this is an introductory course and older 342 participants might already have been trained in these topics. | See also what teachers say about courses without enough practice training in:<br><br>Selvik, S., & Helleve, I. (2022). Understanding Teachers’ Uncertainty in Encounters with Pupils with Experiences of Domestic |

| | | | |
|---|---|---|---|
| | | | Violence. *Scandinavian Journal of Educational Research*, 1-13. |
| | 349-350 | An analysis of Polish participants open-ended responses to the question Training school staff in victimization 16 350 “Any other comments you want to add? Please specify as much as possible” showed that most of them 351 mentioned technical problems (such as the impossibility of watching a linked… | This ought to be mentioned under the data gathering method and analysis.<br>I find this part most suitable under result. |
