# Supplementary material for: How can school help victims of violence? Evaluation of online training for European schools’ staff from a multidisciplinary approach
Source: PLoS One. 2022 Aug 15;17(8):e0272872. doi: 10.1371/journal.pone.0272872 (PMC9377607; doi:10.1371/journal.pone.0272872)
Supplement: Attachment — Submitted filename: Response to reviewers.docx [file pone.0272872.s006.docx]

**Response to the reviewers**

**Comments to the Author**

5. Review Comments to the Author

Reviewer #1: The article approaches an extremely relevant topic for the field of Health and Education. Although the intervention is introductory, it is essential training for education professionals, who often feel helpless and with limited knowledge on the subject. Another strong point of the intervention is the performance of a pilot study, which allows the researcher to make the necessary adaptations for the aim study.

*Response: Thank you very much for highlighting the relevance of our work.*

For each part of the article, there is a complete description of all the elements required for an empirical study, including ethical recommendations. Data analysis is consistent and appropriate to the study, and the combination of qualitative and quantitative data is adequate for this type of investigation, which addresses the issue of sexual violence, a complex phenomenon.

*Response: Thank you very much for your feedback and appreciations.* *Thank you very much as well for the specific comments in the table attached. We included all changes suggested and highlighted modifications in the revised version of the manuscript. Specifically:*

- *Line 42, the sentence was replaced by “As far as children and adolescents are concerned, the developmental victimology theory suggests that children and adolescents are vulnerable to the same forms of victimization as adults (like robbery or assault) and other forms that are specific to their age group related to the dependency from the adult (such as, physical abuse or neglect by their caregivers)”.*
- *Line 91, we included the reference to the mentioned dissertation through the following statement “*It has been shown that sexually abused children who feel safe and communicate effectively with their parents and their protection network tend to believe that adults and the world around them are trustworthy, and therefore may be more prone to disclose their experience of abuse.*”*
- *Line 120, we added further details about the content included in the resilience module.*
- *Line 122: we specified the additional materials available at the end of SAVE course.*
- *Line 530: We included a statement under the limitation section highlighting what you pointed out about recruiting directly children or considering that victims experience may had change over time. The text reads “it should be taken into account as a limitation that what the adult informants tell about their past experiences could have changed, from the moment of their experience to the moment of this investigation. In this sense, we consider that if the informants had been children at the time of the investigation, different results could have been found. So, future investigations may recruit children who have suffered sexual abuse and live in safe conditions during the research, only if the ethical aspects that could challenge such a project can be warranted.”*
- *We would like to clarify that there were “five” European entities members of SAVE (two partners were from Spain), so even if there were four participating countries, there were five institutions. We clarified this in the text, thanks to your comment.*
- *We modified the paragraph starting in line 165 to explain how we dealt with differences in teacher’s training across countries. We also included a statement under the limitation section for readers to consider this aspect when interpreting the results, which reads* “Another important point to consider is that the different training and preparation teachers and school staff must receive in each country may explain some of the findings, and even differences between universities in one region may have influenced the answers of participants.”
- *We moved the reference about conventional content analysis principles to make it clear in which approach we were based to perform our qualitative analysis.*
- *In the limitation section we also mentioned the fact that all participants of Study I were women and from Spain (line 538)*
- *We mentioned the suggested reference (Ellis, 2018), which in fact guided part of our work in line 166.*
- *We included the level of studies achieved by each of the participants in Study I.*
- *In line 310 we detailed all R packages used for the analyses (“In addition to the standard packages, “psych” (Revelle, 2022) and “fmsb” (Nakazawa, 2022) was used to assess psychometric properties, “ggplot2” (Wickham, 2016) to plot analyses. “network” (Butts, 2008; 2015) and “igraph” (Csardi & Nepusz, 2006) to perform and plot network analyses. R Software [60] was used to perform all analyses). We also made the code and dataset available so the study can be reproduced here:* [*https://osf.io/7q3rt/*](https://osf.io/7q3rt/) *(and stated it in line 314).*
- *We modified Figure 2 as you and the editor suggested*
- *We added a paragraph in the discussion about the fact that not all victims perform poorly in school, including the reference you proposed. It states: “Other studies also showed that children might concentrate more on their studies while suffering abuse as a way of coping with the traumatic experience (Selvik, 2020). This is also something brought up by one participant of in Study I, who found in the school a safe place in contrast to what she was living at home. The fact that different victims may cope differently with the abusive experience strengths the need for a real bond between school staff and children, as an effective way to spot potential victims and provide them with help.”*
- *We included a comment about the results of the very recent reference you suggested (Selvik & Helleve, 2022).*
- *We moved the sentence “An analysis of Polish participants open-ended responses to the question “Any other comments you want to add? Please specify as much as possible” showed that most of them mentioned technical problems (such as the impossibility of watching a linked video or the fact that some links took a long time to load). One person highlighted the fact that the available resources were in English and not in Polish, which could have affected their view of the course” to the results section, as suggested.*

# I recommend this article for publication with major revisions.

I have organized my feedback in the following table according to the different parts of the article:

| **Part of the article** | **Line number** | **Citation from the article** | **Comment** |
| --- | --- | --- | --- |
| Abstract |  |  | No comments |
| Introduction | 29 | … forms that are specific to their  age group | Elaborate a bit more on that. What do you mean? Maybe  provide some examples. |
|  | 73-81 |  | I recommend adding to this part the doctoral study of Morrison which is specifically on the disclosure of children who experience sexual abuse:  Morrison, S. E. (2016). *Legal disclosure of childhood sexual abuse: what can professionals tell*  *us?* [Doctoral dissertation]. University of Glasgow. |
|  | 92 | It also proposes a module about resilience… | Can you explain a bit more what you mean with that? This part of information is too short. |
|  | 93 | …and additional material to use in school. | Such as? Could you provide some more information? |
|  | 98-99 | To explore how victims of sexual abuse during childhood remember the school’s role in their lives at the time, to try to find out what helped them and what made it harder for them. | School systems, teacher education, perception of children etc. are in continuous change. What adult informants tell about their past experiences ought to be more problematized and not just taken for granted knowing that the school at informants’ time have changed from the time of their experience to the time of this research.  Its important to discuss this either under method or limitation. It would be also of importance also to discuss what would the research have gained if the informants where to be children at the time of the research, and why not recruiting children who have experienced sexual abuse and were living under safe conditions during the research. |
| Materials and methods | 117 | SAVE was created by a partnership of five European entities based in… | Maybe it should be written four? Since only 4 partnership entities are listed below: Ireland, Italy, Spain,  Poland. |
|  | 124-125 | A challenging aspect of SAVE was that it had to be applicable across Europe, so we had to take into 124 account the particularities of each region, such as the official languages, the different  procedures to 125 | What about the teacher education between these countries?  Teacher education will have a great influence on what and how much teachers know about this topic and how they react. This can be very different between countries so as between universities within the same country. |

|  |  | report child victimization or the required training for school staff members in each country. | Teachers’ attitude and understanding of their role can be of great importance to how they react towards children’s disclosure.  See for example:  Ellis, G. (2018). Containment and denial: Raising awareness of unconscious processes present when teachers are working with children and families where there is domestic  abuse. *Educational Psychology in*  *Practice*, *34*(4), 412–429. |
| --- | --- | --- | --- |
|  | 138 | All of them were Spanish and… | I understand that for convenience informants where all female Spanish.  What about males?  However, would not the research have benefitted from recruiting informants from other parts of Europe also to test out the availability and applicability of this program to European countries other than the countries represented by the 4 partnership entities? And nevertheless, also males?  This ought to be discussed as part of the limitations of this research.  What the age range of the informants of this research? |
|  | 149 | Most were highly educated females. | This says little about the informants.  Are they educated in different fields, or all are graduates from teacher  education? |
|  | 216 | The principles of conventional content analysis… | According to which approach? |
|  | 240 | To store, manage, and organize the data, all transcripts were uploaded to  the Open Source R Software. | Can the authors elaborate more about the use of the R software? |
|  | 269 | All analysis were performed using R | Its unclear. Needs more elaboration. |
| Results | 271-303 |  | Consider another presentation for the results that is more reader friendly. Maybe a table? |
| Discussion | 311 | Previous studies already noted the lower performance… | The authors mention “previous studies” and refers to only one study. However, lower performance it is not always the case. Some children who experience domestic violence might do well at school as a coping strategy. See for example:  Selvik, S. (2020). School strategies of children with multiple relocations at refuges for abused women. *Scandinavian Journal of Educational Research*, *64*(2), 227-241. |
|  | 323 | …importance of a private, safe space  to share their feelings… | As Morrison (2016) report in her research “the opportunity to tell”. |
|  | 341-342 | It could also be due to the fact that this is an introductory course and older 342 participants might already have been trained in these  topics. | See also what teachers say about courses without enough practice training in:  Selvik, S., & Helleve, I. (2022). Understanding Teachers’ Uncertainty in Encounters with Pupils with  Experiences of Domestic |

|  |  |  | Violence. *Scandinavian*  *Journal of Educational Research*, 1-13. |
| --- | --- | --- | --- |
|  | 349-350 | An analysis of Polish participants open-ended responses to the question Training school staff in victimization 16  350 “Any other comments you want to add?  Please specify as much as possible” showed that most of them 351 mentioned technical problems (such as the impossibility of  watching a linked… | This ought to be mentioned under the data gathering method and analysis.  I find this part most suitable under result. |

Below I present my suggestions:
Instruments:
In the Study I, the authors mention that the participants were also asked about several characteristics of the course content and they were invited throughout the interview to make comments or suggestions. I suggest that the authors inform at what point in the research these participants had access to the course content. I also suggest they make it clearer that this study analyses one module of the intervention related to sexual abuse (before the presentation of the goals).

*Response: Thank you very much for these suggestions. We modified the Procedure section to clarify when participants were presented with the content of the course. We also included the specific aim of assessing the sexual victimization module through the opinion of victims of sexual abuse.*

Discussion:
In the first paragraph of the discussion, the authors wrote that the “the present study analyses the views of education staff and female victims of child sexual abuse on the role of the family and school environment in child protection”, different from abstract and introduction. I suggest that the authors standardize the aim of the study.

*Response: Thank you. We modified the phrase (line 401) and we restructured the discussion section linking each study results with the aims proposed.*

On line 308, I suggest that the authors inform that the discussion refers to Study I as they wrote at the beginning of lines 337 (Study II), and 366 (Study III).

*Response: Thank you very much for your feedback, we reformulated the main objective in the discussion and added “Study I” in the text, which makes the article way more clear and easier to read.*

In the data analysis, the authors mentioned that they extracted 74 fragments (I couldn't check because table 4 is not available for my viewing). However, few results were discussed, especially about the impact of the course (only one paragraph). I suggest that the authors explore this data further in a more in-depth analysis.

*Response: Thank you again. We have now correctly included the tables within the text. We agree that currently the text corresponds better to the data. We decided not to go further on the analyses as we focused only in our main findings.*

Figure and Tables:
- I didn’t have access to the Figure A1, which was provided as supplementary material.
- I didn’t have access to the Tables 2, 3, 4, and 5. Also it is important to mention where the tables 2, 3, and 4 need to be included in the text.

*Response: Thank you very much for pointing this out. We included now the tables in the same document where they should be placed. We also updated the supplementary material and included all supporting information in* [*https://osf.io/7q3rt/*](https://osf.io/7q3rt/)

In view of this, I recommend that the authors make the necessary adjustments for the publication of the text.

*Response: Thank you once more for helping us to improve our work.*

Reviewer #2: Thank you for an interesting article. The idea of your article is of great importance knowing the number of digital courses and online resources that are developed to support teachers in their encounters with children experiencing violence without being evaluated. We therefore do not know much about these programs’ effectiveness, availability for teachers, and to what extend they meet the teachers’ and the victims’ needs. Your design is creative and well explained.

*Response: Thank you very much for your positive feedback.*

The article would benefit from a theoretical framework to provide a deeper analysis of the data and a more thorough discussion. For the meantime, the discussion part is more or less descriptive than discussive.

*Response: Thank you very much for your suggestion. We have framed our work within the basis of the theoretical framework developed by Prof. Finkelhor's, i.e., Developmental victimology. We have also modified the discussion and introduction including new references pointed out by other reviewers, which we agree that enriched the framework and the arguments.*

Reviewer #3: This interesting work focuses on a topic of interest in the areas of health, education and psychology, and has the following objectives: to analyze how victims of sexual abuse during childhood remember the school's role in their lives at the time, to try to find out what helped them and what made it harder for them; to assess the potential of an online training course (SAVE) developed at European level, through the feedback given by school staff members and victims of child sexual abuse; to assess schools’ potential in the prevention, detection and reporting of child victimization through changes that the staff of educational centers would include in their everyday practice as a result of the course; and to test to what extent the actions proposed by school staff members meet the needs described by victims regarding the school's role when they suffered child sexual abuse. The online course used and called SAVE was created by a partnership of five European entities, which gives an important packaging to the contribution of this research.

*Response: Thank you very much for your careful appreciation of our work.*

In general, the article is well written and articulated. The review of the scientific literature carried out by the authors to support the work seems correct and pertinent to me, and at the same time I consider that the theoretical introduction is of quality, although I recommend that previous teacher training and intervention programs be reviewed in greater depth in matters of maltreatement and abuse, because there are very interesting contributions both nationally and internationally that are not named in the article.

*Response: Thank you for you this suggestion. We included more studies about teacher trainings and intervention programs (e.g., Gün et al., 2022). We also referred to a meta-analytic review about the components of school programs that successfully tackle child abuse (Gubbels et al., 2021).*

The description of the methodological part is complete regarding the procedure. The authors could add more data about the samples and the instrument used.

*Response: Thank you very much for these suggestions. We also added further details about the sample of study I (age range, level of studies achieved) and study II (level of studies achieved, professional profile). We also made all instrument protocols available and specified some details (administration platform, item wording) in the instrument section.*

The results are well structured and interesting. It is very commendable that authors have been able to articulate three studies to give a joint response to the objectives of interest. However, and unfortunally, I could not accurately evaluate this section since in the information offered to me are missed the results Tables. The figure that is presented must be improved so that its visualization is more comprehensive and letters and figures do not overlap.

*Response: Thank you very much for your comments. We modified the figure and included the tables in the same manuscript document. We are sorry that you were not able to review this material and hope you find it relevant.*

The discussion is also well articulated. The authors could plan to formulate clear hypotheses and organize the discussion around them. Future lines of research are appreciated. It is recommended that the concrete contribution and practical application of the work be highlighted.

*Response: Thank you very much for the suggestion. We included more specific information about the contributions of this research and propose new lines of research for the future. Regarding the organization of the discussion, we agree it could be better presented. Instead of making new hypotheses, we decided to organize the discussion around the objectives described at the beginning of the investigation. We believe the text is now clearer and more coherent.*
